# Discovery and characterisation of an antibody that selectively modulates the inhibitory activity of plasminogen activator inhibitor-1

**DOI:** 10.1038/s41598-019-38842-x

**Published:** 2019-02-07

**Authors:** Katherine A. Vousden, Tomas Lundqvist, Bojana Popovic, Brian Naiman, Alan M. Carruthers, Philip Newton, Daniel J. D. Johnson, Anja Pomowski, Trevor Wilkinson, Patrick Dufner, Isabelle de Mendez, Philip R. Mallinder, Clare Murray, Martin Strain, Jane Connor, Lynne A. Murray, Matthew A. Sleeman, David C. Lowe, James A. Huntington, Tristan J. Vaughan

**Affiliations:** 1 MedImmune Ltd, Cambridge, CB21 6GH UK; 2 AstraZeneca AB R&D, Pepparedsleden 1, 431 50 Mölndal, Sweden; 3 MedImmune LLC, One MedImmune Way, Gaithersburg, MD 20878 USA; 4 Department of Haematology, Cambridge Institute for Medical Research, University of Cambridge, Cambridge, CB2 0XY UK; 5 AstraZeneca R&D, Alderley Park, Macclesfield, Cheshire, SK10 4TF UK

## Abstract

Plasminogen activator inhibitor-1 (PAI-1) is a serine protease inhibitor (serpin) that regulates fibrinolysis, cell adhesion and cell motility via its interactions with plasminogen activators and vitronectin. PAI-1 has been shown to play a role in a number of diverse pathologies including cardiovascular diseases, obesity and cancer and is therefore an attractive therapeutic target. However the multiple patho-physiological roles of PAI-1, and understanding the relative contributions of these in any one disease setting, make the development of therapeutically relevant molecules challenging. Here we describe the identification and characterisation of fully human antibody MEDI-579, which binds with high affinity and specificity to the active form of human PAI-1. MEDI-579 specifically inhibits serine protease interactions with PAI-1 while conserving vitronectin binding. Crystallographic analysis reveals that this specificity is achieved through direct binding of MEDI-579 Fab to the reactive centre loop (RCL) of PAI-1 and at the same exosite used by both tissue and urokinase plasminogen activators (tPA and uPA). We propose that MEDI-579 acts by directly competing with proteases for RCL binding and as such is able to modulate the interaction of PAI-1 with tPA and uPA in a way not previously described for a human PAI-1 inhibitor.

## Introduction

Plasminogen activator inhibitor 1 (PAI-1) is a member of the serine protease inhibitor (serpin) superfamily^1^ and is an important therapeutic target for coronary thrombosis, as well as fibrotic diseases and many cancers^2,3^. The major physiological role of PAI-1 is to block the conversion of plasminogen to plasmin by tissue-type plasminogen activator (tPA) and urokinase-type plasminogen activator (uPA)^4^. PAI-1 is also a key modulator of cell adhesion and motility through blocking vitronectin binding to integrins^5^, a function wholly independent of its protease inhibition role^6^. Crystal structures of PAI-1 in complex with uPA^7^, tPA^8^ and vitronectin^9^ have been solved, revealing that these interactions occur in spatially distinct parts of the molecule.

**Figure 1:**
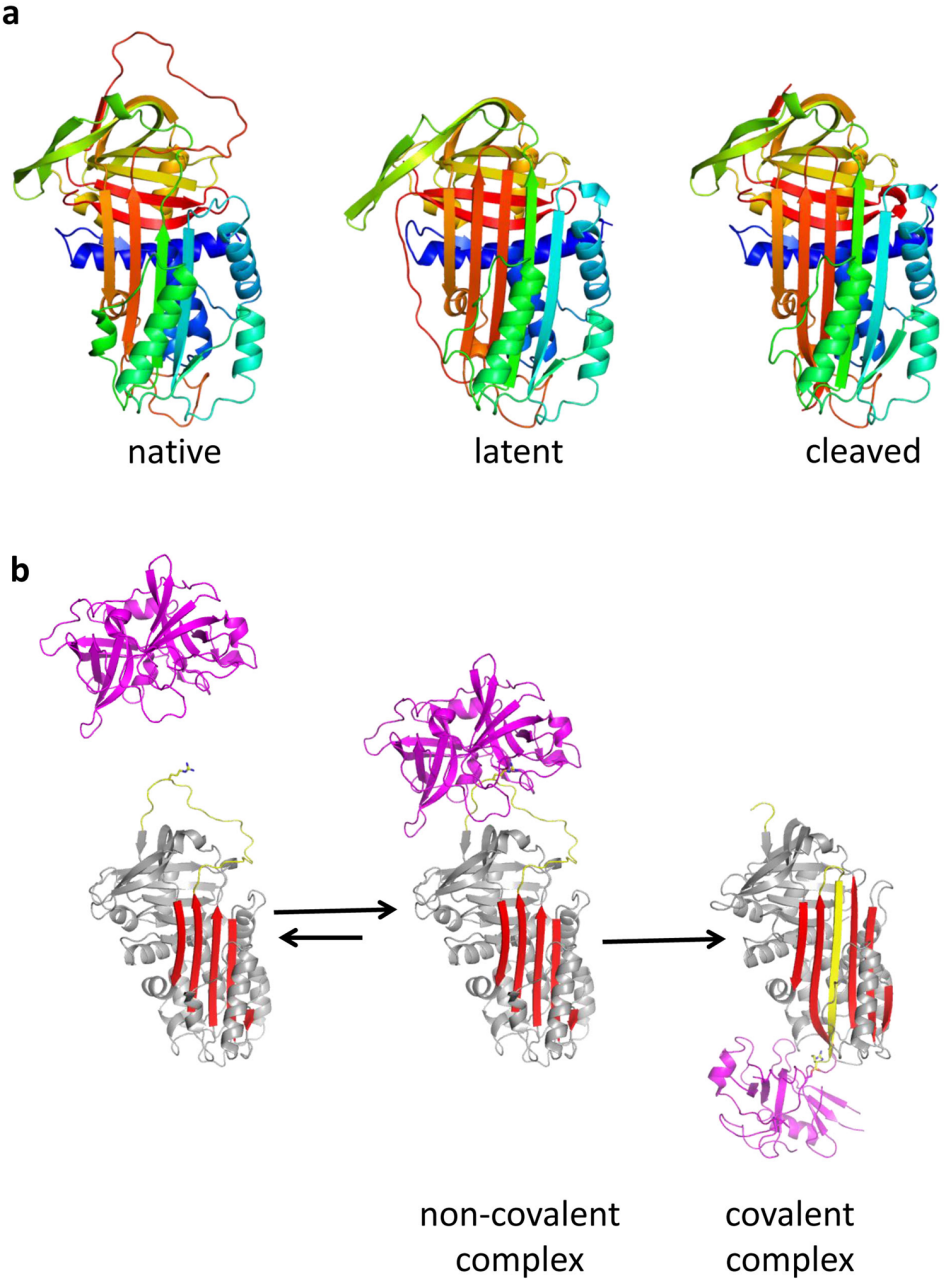
Structural forms of PAI-1 and the serpin mechanism of protease inhibition: (**a**) PAI-1 is a conformationally labile protein and can rapidly transition from the native (left, 3pb1^7^) to the latent (middle, 1lj5) state. Ribbon diagrams are shown coloured from N-to-C terminus (blue to red). Conversion to the latent state involves incorporation of the RCL (loop at top) into β-sheet A (front sheet) and the extension of strand 1 of β-sheet C (s1C). As with most serpins, as similar conformation is obtained upon cleavage within the RCL (right, 3cvm^58^). (**b**) Mechanism of protease inhibition by PAI-1 depicted using PDB structures 5brr^8^ (tPA:PAI-1) and 1ezx^59^ (anti-trypsin:trypsin). The elements of PAI-1 responsible for protease inhibition are the RCL (yellow, with P1 Arg depicted as sticks) and β-sheet A (red). After recognition of the RCL by a protease (magenta, centre), the protease is irreversibly translocated to the opposite pole of PAI-1 and trapped as a covalent complex (right).

PAI-1 exhibits profound conformational plasticity with native (or active), latent and cleaved conformations reported (Fig. 1a), and an additional ‘substrate’ conformation proposed^10–13^. PAI-1 is synthesised in the active conformation, which is characterised by the accessibility of its reactive centre loop (RCL) to protease binding^12,14^. The RCL (designated P17 to P3′) includes a ‘bait’ peptide bond (P1-P1′) that mimics the normal substrate of the target proteases^13^. The number after ‘P’ indicates the position of the residue N-terminal to the scissile bond; the prime indicates residues C-terminal to the scissile bond. Interaction of this bait region with the active site of either tPA or uPA in a 1:1 stoichiometric complex results in cleavage of the P1-P1′ bond and extensive structural re-arrangement, characterised by the insertion of the N-terminal portion of the RCL into β-sheet A and the complete translocation of the protease to the opposite pole of the PAI-1 molecule (Fig. 1b). The PAI-1:protease complex is stable and results in both the inhibition of protease and the inactivation of PAI-1. PAI-1 can also act as a substrate if protease translocation is slowed by the binding of certain ligands^11,15^.

PAI-1 is unique amongst the serpins because of its ready conversion from the native to the latent state. The half-life of native PAI-1 is approximately 2 hours at 37 °C *in vitro*, and is slightly longer *in vivo* due to the high-affinity association with the somatomedin domain of vitronectin. Inhibitory activity is dependent on the exposure of the RCL in the native state, so the latent form is unable to inhibit proteases. The P1-P1′ bond is also inaccessible to proteolytic attack in the latent conformation^12^.

Work with both neutralising antibodies and small molecule inhibitors have elucidated multiple mechanisms of action for the prevention of the initial non-covalent Michaelis-Menton complex formation between PAI-1 and its target serine proteases. Two of these mechanisms are irreversible: the accelerated conversion of active PAI-1 to latent and the conversion of active PAI-1 to a substrate form^16–18^. Both these mechanisms act by altering the kinetics of RCL insertion^19,20^. A reversible mechanism of action has also been described, which involves modulation of RCL conformation to prevent serine protease binding^21^. In addition, inhibitors that prevent Michaelis-Menton complex formation may also inhibit or affect the kinetics of PAI-1 binding to vitronectin^22^. The key to developing therapeutic compounds is to understand the specific mechanism of action by the investigational drug on the target within a particular disease setting. However, despite a wealth of *in vitro* characterisation on selective PAI-1 inhibitors, there is a deficiency in the reporting of *in vivo* data to link the relevance of mechanistic findings to real pharmacological effects. This is often due to a lack of rodent cross-reactivity, or impaired binding to endogenous glycosylated PAI-1^23^.

Epitope mapping of PAI-1 antibody inhibitors is a crucial step towards linking specific molecular mechanisms of PAI-1 blockade with *in vivo* pharmacology. Neutralising PAI-1 antibody epitopes have been derived by alanine scanning^22^, the use of chimeric proteins^24^ or by combining *in silico* docking with site-directed mutagenesis^25^. To date, no crystal structure of PAI-1 in complex with an inhibitory antibody has been reported. The multiple structural conformations that PAI-1 can adopt, and the inherent flexibility and short half-life of the active form pose challenges to X-ray crystallography.

Here we describe the *in vitro* and *in vivo* characterisation of neutralising anti-PAI-1 antibody MEDI-579 and the crystal structure of MEDI-579 Fab in complex with human PAI-1. The structure explains the unique characteristics of MEDI-579: inhibition of PAI-1 anti-protease activity but not vitronectin binding; specificity for active compared to latent PAI-1; and rodent PAI-1 cross-reactivity. The cross-species reactivity of MEDI-579 enabled the specific contribution of PAI-1 anti-protease activity to be characterised in a disease-relevant model of fibrosis. Our data directly combine the first structural study of a neutralising PAI-1 antibody with its *in vivo* pharmacological effects and provides a structural framework for understanding the consequence of specific blockade of protease binding.

## Results

### Generation and *in vitro* characterisation of recombinant antibody MEDI-579

**Figure 2:**
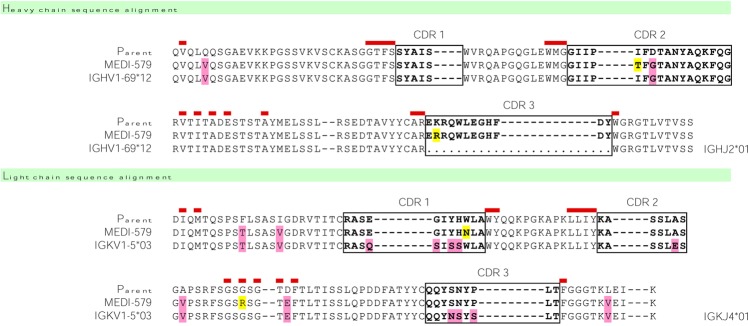
MEDI-579 sequence. MEDI-579 is shown in alignment with parent sequence and closest human germline matches. CDR sequences are boxed. Vernier residues are marked in red above the sequence. Pink shading in MEDI-579 represents positions reverted to match closest germline. Yellow shading in MEDI-579 indicates positions mutated from parent during affinity maturation.

A panel of neutralising PAI-1 specific, human, rat and mouse cross-reactive antibodies were isolated from a large phagemid–based library of single-chain antibody variable fragments (scFv)^26,27^, by panning on recombinant human and rat PAI-1 followed by screening in a PAI-1/tPA competition binding assay. A scFv was selected for affinity maturation by ribosome display *in vitro* evolution^28,29^. Selection cycles were performed using either human PAI-1 alone, or by alternating rounds of selection on human and rat PAI-1. High-throughput screens using human and rat PAI-1 were run in parallel to identify scFv variants with improved activity across both species. Significant rodent cross-reactivity was only achieved when both human and rat antigens were utilised in the selection and screening campaign (Supplementary Fig. S1). No human-rat cross-reactive scFvs were isolated from selections on human PAI-1 alone. The most potent scFv, Ab167, was able to neutralise recombinant human, rat and mouse PAI-1 activity in species-relevant tPA-coupled chromogenic assays (Supplementary Fig. S1). To reduce potential immunogenicity during clinical development, four variable heavy (VH) and five variable light (VL) chain framework residues were reverted to the corresponding residues from their closest human germline sequences (IGHV1-69*12 and IGKV1-5*03 for VH and VL chains respectively) to generate MEDI-579 (Fig. 2).

The fully human antibody, MEDI-579 was formatted as both Fab and human IgG_1_ and subjected to extensive characterisation.

To determine if MEDI-579 could bind to the latent form of PAI-1, a competition ELISA experiment was conducted. The commercial preparation of latent human PAI-1 was able to efficiently compete with native for binding to MEDI-579, but with an apparent 10-fold lower affinity (Supplementary Fig. S2). However, the results were also consistent with a ~10% contamination with native PAI-1, perhaps from incomplete conversion to the inactive latent form. This was addressed by incubating an excess of MEDI-579 with the latent material and running the complex on a size exclusion column (Supplementary Fig. S2). Most of the material did not form complex with MEDI-579 and eluted as monomeric PAI-1. A small high molecular weight peak contained the rest of the PAI-1. Densitometry analysis of an SDS gel revealed that 12.5% of the PAI-1 complexed with MEDI-579, while the remaining 87.5% was unable to interact with the antibody. Moreover, this uncomplexed PAI-1 fraction had no activity in a thrombin cleavage assay (Supplementary Fig. S2). We concluded that the conversion to latent was incomplete and that the competition ELISA results reflected a small but significant contamination with native PAI-1.

**Figure 3:**
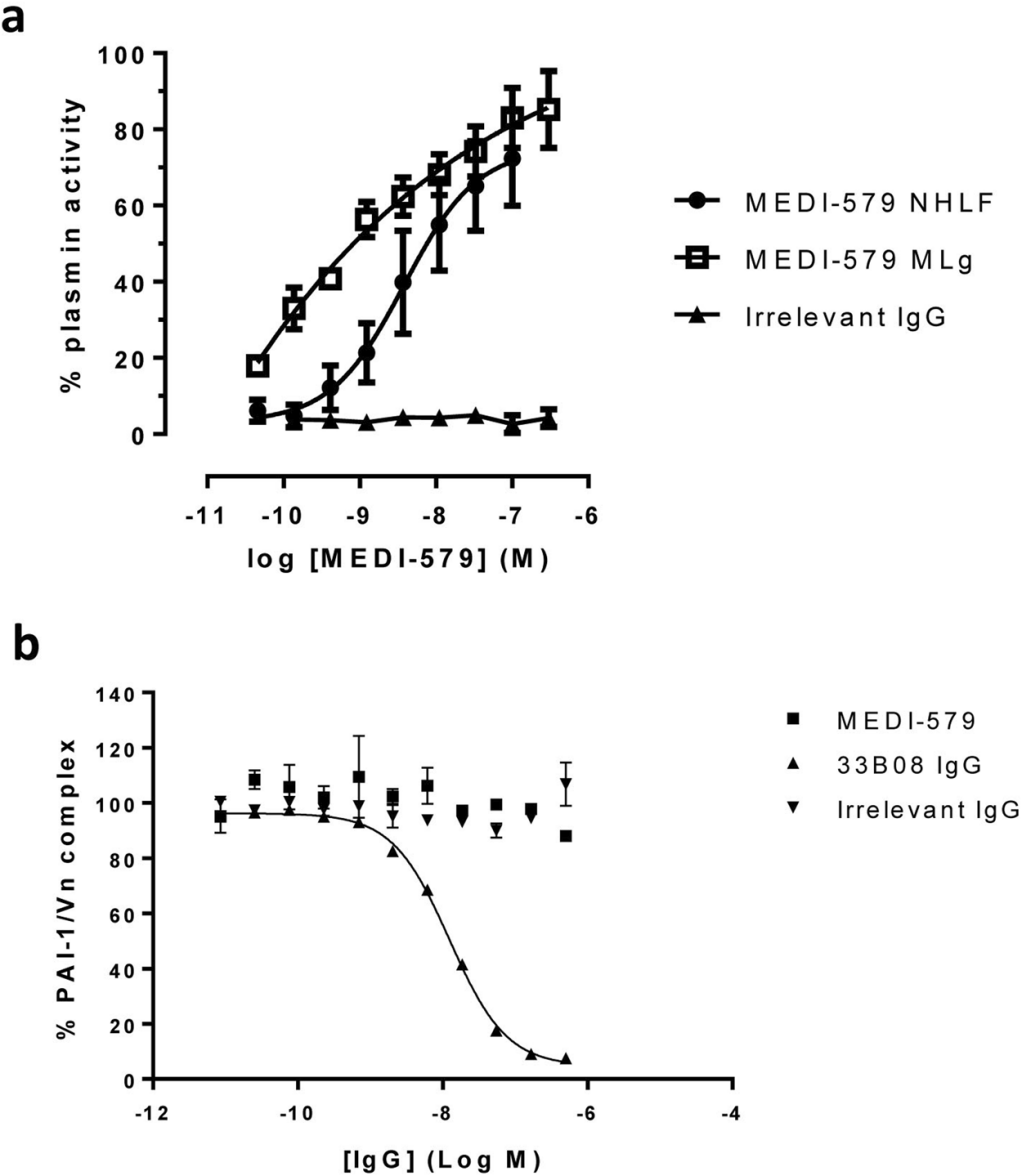
*In vitro* characterization of MEDI-579 (**a**) TGFβ-1 stimulated fibroblasts produce PAI-1. The ability of this endogenously produced PAI-1 to inhibit protease in an assay dependent on the generation of active plasmin from exogenously added plasminogen is then measured. Cells with (+PAI-1) or without (-PAI-1) TGFβ-1 stimulation represent 0% or 100% plasmin activity respectively. MEDI-579 is able to inhibit PAI-1 produced from TGFβ-1 treated normal human lung fibroblasts (NHLFs) and mouse lung fibroblasts (MLgs) in this assay with pIC_50_ = 9.8 ± 0.14 (10.5 nM) and 8.99 ± 0.12 (1.3 nM) respectively. Data shown are an average of four independent experiments ± s.e.m. (**b**) MEDI-579 does not block PAI-1 binding to vitronectin. Antibody 33B08 enhances conversion of active PAI-1 to the latent form^16^ and potently inhibits in this assay (IC_50_ = 12.46 ± 0.45 nM; data is average of two experiments ± s.d.).

It has previously been demonstrated that glycosylation can have a profound effect on the inhibitory activity of anti-PAI-1 antibodies^23^. To demonstrate that MEDI-579 is able to inhibit pharmacologically relevant PAI-1, MEDI-579 IgG_1_ was tested for functional blockade of endogenous PAI-1 released from TGFβ1-stimulated normal human lung fibroblast (NHLF) and mouse lung fibroblast (MLg) cells^30^. MEDI-579 was able to inhibit plasminogen activation by endogenous PAI-1 with pIC_50_ values of  9.8 ± 0.14 (IC_50_ 10.5 nM) and 8.99 ± 0.12 (IC_50_ 1.3 nM) in human and mouse assays respectively (Fig. 3a). The level of other endogenous components of these species-specific plasminogen systems are undefined in these assays, so IC_50_ values cannot be compared to give relative measures of affinity. MEDI-579 does not block PAI-1 binding to vitronectin (Fig. 3b), demonstrating that MEDI-579 specifically acts to prevent protease binding to PAI-1. Competition binding experiments also demonstrates that MEDI-579 does not interact with other circulating serpins (Supplementary Fig. S3).

The dissociation constant (K_D_) of MEDI-579 Fab for human PAI-1 was evaluated by BIAcore. MEDI-579 is an ultra-high affinity antibody, with an off-rate constant (k_d_) in the order of 4×10^−5^ s^−1^ and a K_D_ of approximately 6 pM. MEDI-579 Fab also binds rat PAI-1 with high affinity by this method (Table 1; Supplementary Fig. S4).

**Table 1:** MEDI-579 affinity data.

	ka (1/Ms)	kd (1/s)	KD (pM)	Replicates
Human PAI-1	7.02E + 06 (±9.70E + 05)	4.18E−05 (±1.52E − 05)	6 (±2)	n = 6
Rat PAI-1	7.64E + 06 (±5.21E + 05)	7.97E−04 (±1.76E − 04)	105 (±30)	n = 2

BIAcore analysis was performed on MEDI-579 Fab binding to recombinant PAI-1. Data are averages ± standard deviation of replicates as indicated.

### MEDI-579 treatment inhibits active PAI-1 and prevents the development of proteinuria and pathological changes in the kidney in a mouse model of lupus nephritis

MEDI-579 specifically inhibits serine proteases binding to PAI-1, while binding to vitronectin is retained. PAI-1 expression is increased in animal models of fibrosis^31,32^ and genetic deletion of PAI-1 has been shown to be protective^33–35^.

The role for PAI-1 in glomerulonephritis is well established and has been reported to be independent of vitronectin^36^. We evaluated the ability of MEDI-579 to inhibit PAI-1 in a mouse model of lupus nephritis^37^ to determine whether MEDI-579 is able to provide protection from the development of glomerulonephritis.

**Figure 4:**
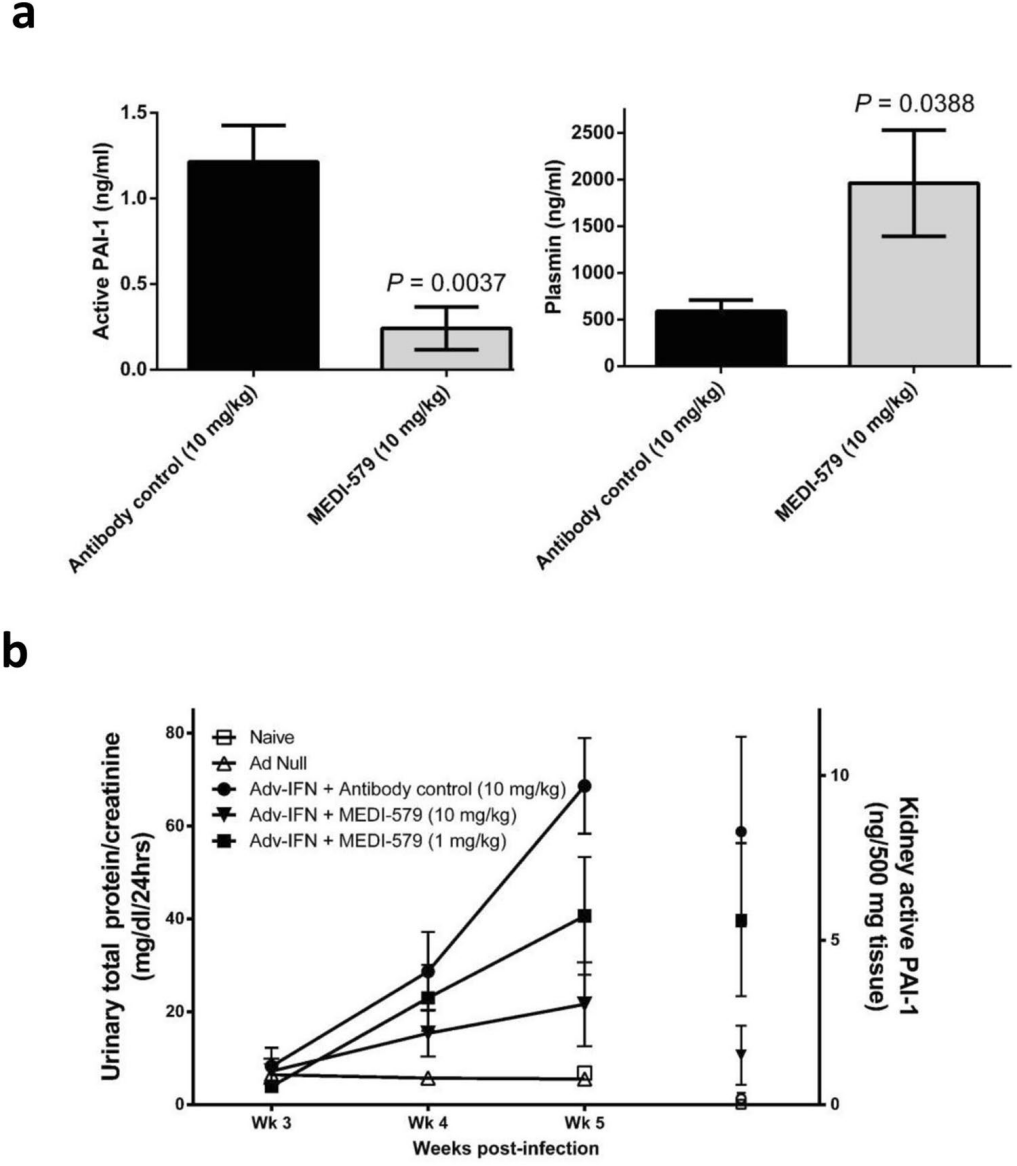
MEDI-579 treatment in adenovirus-interferon-alpha accelerated kidney disease in NZBW/F1 mice. (**a**) Plasma levels of active PAI-1 and Plasmin 48 hr following a single dose of either antibody control or MEDI-579, n = 5 mice/group, shown as group average ± SEM; P = 0.0037 (active PAI-1) and P = 0.0388 (plasmin) by unpaired one-tailed t test with Welch’s correction. (**b**) MEDI-579 dose-dependently reduces urinary total protein (normalised to creatinine); *P < 0.05 MEDI-579 (10 mg/kg) compared to antibody control at Wk 5, Kruskal-Wallis with Dunn’s multiple comparison. PAI-1 levels in kidney homogenates are also shown; *P < 0.05 MEDI-579 (10 mg/kg) compared to antibody control (10 mg/kg), Kruskal-Wallis with Dunn’s multiple comparison post-tests.

A single dose of 10 mg/kg MEDI-579 inhibited circulating active PAI-1 and elevated plasmin 48 hours following administration in this model (Fig. 4a**)**. Treatment with MEDI-579 for three weeks provided dose-dependent protection from the development of proteinuria (Fig. 4b) and decreased urinary sodium output, another measure of renal function (Supplementary Fig. S5). This dose-dependent protection of kidney function correlated with inhibition of active PAI-1 levels in both plasma and kidney homogenates (Fig. 4b).

### MEDI-579/PAI-1 structural determination

**Figure 5:**
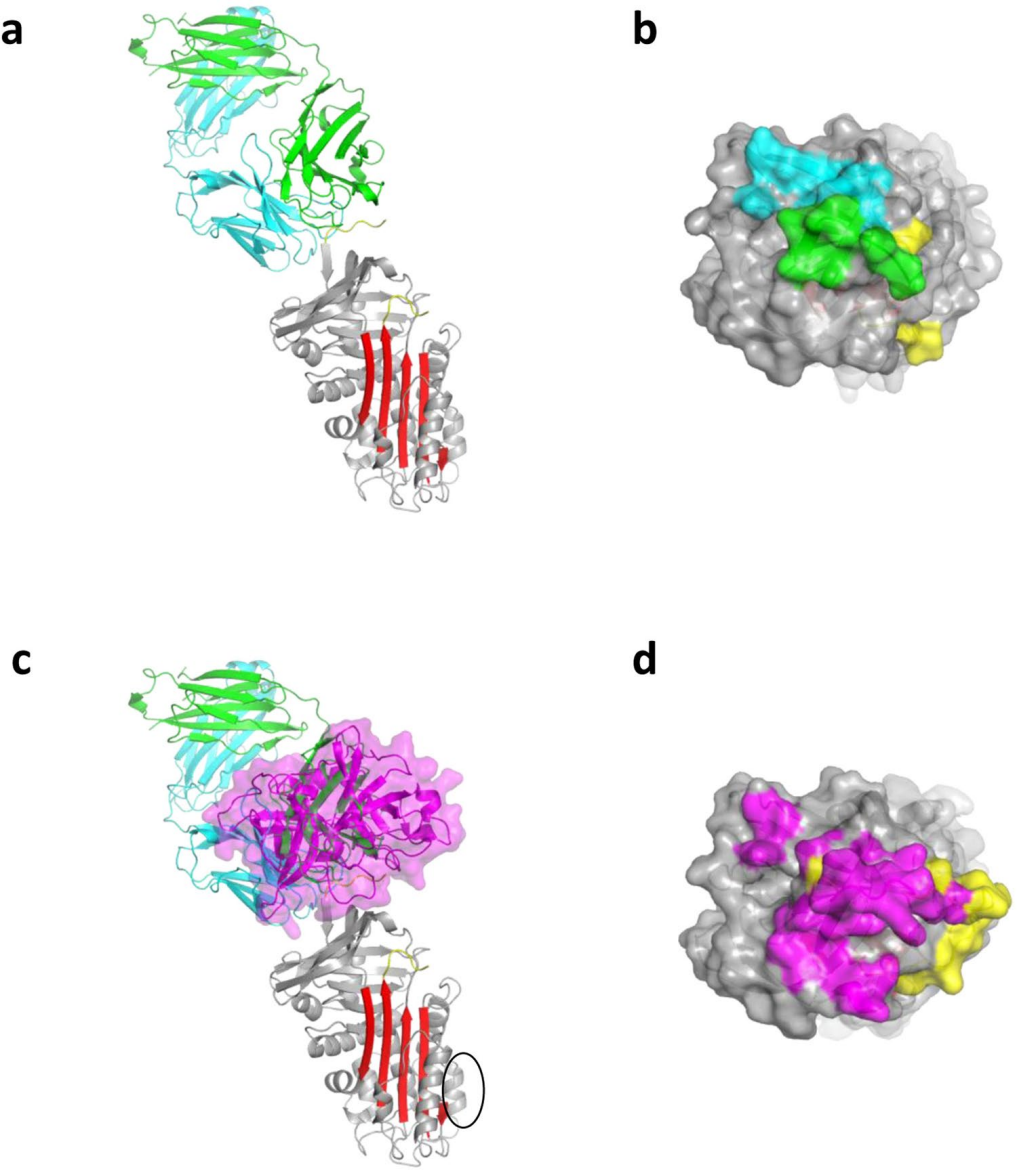
Crystal structure of MEDI-579 Fab/PAI-1 complex. (**a**) A ribbon diagram of a representative structure of the Fab-PAI-1 complex is show. MEDI-579 Fab (heavy chain in green, light chain in cyan) binds in a ‘protease-like’ manner, interacting with the RCL (yellow) and adjacent sites. (**b**) A surface representation of the top of PAI-1 with the RCL in yellow and the contact regions of the heavy and light chains coloured green and cyan, respectively, illustrates the footprint of the Fab on PAI-1. (**c**) The same ribbon depiction of the Fab/PAI-1 complex with the position of uPA from 3pb1^7^ illustrates the overlap of the binding sites. The reported vitronectin binding site on PAI-1 is indicated by a black circle. (**d**) The surface of the top of PAI-1 is coloured as in panel (**b**), but with the uPA footprint in magenta.

The crystal structure of the complex between PAI-1 and MEDI-579 Fab was determined by molecular replacement and refined to an *R*-factor of 22% and an R_free_ of 24% (Table 2). The asymmetric unit is composed of four PAI-1:Fab complexes, denoted AIE, BJF, CKG and DLH for PAI-1 and the light and heavy chains of the Fab, respectively. The complexes are essentially identical, but AIE was chosen for analysis due to its lower average B-factors. With the exception of the RCL, the conformation of PAI-1, including within the reported vitronectin binding site, is unaltered by its interaction with MEDI-579 Fab with an RMSD of 1 Å when compared to uncomplexed native PAI-1 (3q03^38^). MEDI-579 Fab binds to the ‘top’ of PAI-1 directly engaging the RCL, including the scissile P1-P1′ residues, and an exosite immediately adjacent to the RCL (Fig. 5a,b). The interface buries a total of 1745 Å^2^, with the majority (60.5%) involving the light chain of the Fab. Interactions with the RCL, from P2 to P3′, accounts for 35% of the total buried surface area, with almost all of the contacts (91%) involving the Fab heavy chain. The interaction between the heavy chain of the Fab and the RCL of PAI-1 is sufficient to explain both the ability of MEDI-579 to inhibit PAI-1-protease interactions and the lack of binding to latent PAI-1. However, for the two main targets of PAI-1, uPA and tPA, a conserved exosite adjacent to the RCL is also used for recognition. Both uPA and tPA place an Arg residue (Arg37A - chymotrypsin numbering) onto a negatively-charge surface on β-sheet C of PAI-1 to make a salt-bridge with Glu212. The MEDI-579 Fab similarly contacts this site using VL chain residues His31 and Arg66. Therefore, MEDI-579 inhibits PAI-1 by blocking both the RCL and the exosite region necessary for recognition of its two targets uPA and tPA. The overlap of binding sites on PAI-1 is illustrated in Fig. 5c,d. The position of the Fab on PAI-1 is remote from the reported vitronectin binding site (Fig. 5c), which is unaltered upon Fab binding. This supports our observation that binding to vitronectin is preserved in the presence of MEDI-579 (Fig. 3b)^9^, although subtle changes in binding affinity due to allosteric or other effects cannot be ruled out.

**Table 2:** Data and refinement statistics.

	ID29 ESRF
**Data collection**	
Space group	P2_1_
Cell dimensions	
*a*, *b*, *c* (Å)	90.2 89.6 249.7
*a*, *b*, *g* (°)	90.0 99.3 90.0
Osc./# frames	0.2/1100
Wavelength	0.976
Resolution (Å)	15–2.9 (3.1–2.9)
*R*_sym_ or *R*_merge_	0.095 (0.47)
*I*/s*I*	4.8 (1.6)
Completeness (%)	98.9 (99.1)
Redundancy	4.4 (4.4)
**Refinement**	
Resolution (Å)	2.9
No. reflections	86268
*R*_work_/*R*_free_	0.22/0.24
No. atoms	
Protein	24572
Ligand/ion	—
Water	46
B-factors	
Protein	63.1
Ligand/ion	—
Water	41.6
R.m.s deviations	
Bond lengths (Å)	0.010
Bond angles (°)	1.12

The paratope of MEDI-579 is defined by the twenty-five amino acid residues within 4.0 Å of the sixteen amino acids of the PAI-1 epitope (Table 3). The paratope includes residues Arg66, Ser67 and Gly68 are within part of a light chain framework region that forms an outer loop, distinct from the CDR loops. Two of these residues, VL Arg66 and Gly68, are Vernier residues and known to be important for antibody structural integrity. During affinity maturation, the appearance of an arginine in place of the glycine at position VL66 was linked to an increase in rodent cross-reactivity. In the structure, MEDI-579 VL Arg66 forms a stacking interaction with human PAI-1 Tyr220, and is predicted to form a salt-bridge with the negatively charged Glu220 present at this position in rodent PAI-1 (Supplementary Fig. S6a). This position is one of three sequence differences between rodent and human PAI-1 mapped within the epitope (human positions first): Tyr220Glu, Tyr241Phe and Glu350Thr. These amino acid substitutions are identical in mouse and rat PAI-1 sequences (Supplementary Fig. S6b). The Tyr241Phe substitution would be predicted to have a relatively neutral effect on the binding energy of the interface, as the pi-pi stacking interaction with His31 in VL CDR1 observed in the crystal structure would be preserved. Glu350 however appears central in a network of polar interactions as it engages charged residues from both the heavy and light chains: Arg97 in VH CDR3 and Lys50 in VLCDR2. A Glu350Thr substitution at this position would disrupt this network and contribute towards the overall loss in affinity still observed for MEDI-579 between human and rodent PAI-1 species (Supplementary Fig. S6c). In support of the key role predicted for MEDI-579 VL Arg66 in the rodent PAI-1 interaction, it was demonstrated that reversion of Arg66 back to Gly66 in Ab167 scFv reduced binding to rat PAI-1 by approximately 300-fold while binding to human PAI-1 was not significantly altered (Supplementary Fig. S7).

**Table 3:** MEDI-579 paratope residues.

VH chain^a^	Residue^b^
CDR2	Gly 50, Iso 51, Iso 52, Phe 54, Thr 56, Ala 57, Asn 58
CDR3	Glu 95, **Arg 97**, Trp 99, Leu 100, Glu 100a, Gly 100b
**VL chain** ^**a**^	
CDR1	Tyr 30, His 31, Asn 32*
CDR2	**Lys 50**
FW3/outer loop	Arg 66*, Ser 67, Gly 68
CDR3	Tyr 91, Ser 92, Asn 93, Tyr 94, Leu 96

Residues within 4.0 Å of PAI-1 are listed and categorised by Kabat definition. Residues involved in H-bonding networks are highlighted in underline. Residues that form salt-bridges with PAI-1 are highlighted in bold. *Indicates residues introduced during affinity maturation.

## Discussion

Serpins play a key role in controlling a diverse array of proteolytic pathways central to human health. Members of the serpin family are defined by their highly conserved core structure, including the presence in each of a reactive centre loop which is used as bait for specific protease capture. Once bound to the bait peptide, the protease/serpin complex undergoes a huge conformational change (for review see Huntington, 2011)^39^. This conformational flexibility makes targeting of serpins for therapeutic effect and understanding the mechanistic and pharmacological consequences of serpin inhibition challenging. The central role of PAI-1 in many disease processes makes it an attractive target for drug development. However, despite an interest in targeting this serpin, there are no PAI-1 inhibitors clinically approved in any indication^40^.

Here, we report the isolation and affinity maturation of a highly potent neutralising antibody to PAI-1 and the crystallisation of the PAI-1/MEDI-579 Fab complex. MEDI-579 interactions across the RCL and a key protease exosite supports a directly competitive mechanism of action for this antibody and rationally explains the observed specificity of MEDI-579 for the active conformation of PAI-1, and the specific blockade of protease, but not vitronectin, binding. The rodent cross-reactivity that enabled the *in vivo* pharmacology was achieved through a rationally designed antibody engineering, selection and screening cascade. From the paratope analysis enabled by the structure, one mutation introduced during optimisation (VL Arg66) can be identified as a key contributor to the significant increase in rodent cross-reactivity observed. This residue is in the light chain outer loop, in a framework region not usually targeted for antibody engineering. Our strategy of introducing random mutations across the whole V-region and then affinity selection of these libraries by ribosome display using iterative selection cycles on human and rodent PAI-1 was successful in identifying this key solution to significantly improving rodent cross-reactivity. The importance of the heavy chain outer loop in antigen binding is well studied, and is reported to account for 1.3% of human antibody-antigen contacts^41,42^. To our knowledge, this is the first report of a light chain outer loop residue being modified during an *in vitro* affinity maturation campaign to play a direct role in antibody function.

In addition to its role as the primary inhibitor of plasminogen activators, PAI-1 also regulates cell migration and adhesion through competition with the receptor for cell bound urokinase-type plasminogen activator (uPAR) and integrin αVβ3 for vitronectin binding^5,43^. To better realise the therapeutic potential of PAI-1 blockade, it is important to understand the relative contribution of these two pathways in each disease setting. Studies reported to define the contribution of vitronectin and/or protease binding roles of PAI-1 in fibrotic or angiogenic disease pathways have been limited to the administration of recombinant mutant PAI-1 molecules^44–46^, which may be impacted by the presence of endogenous PAI-1 or challenges in administering PAI-1 at physiologically relevant levels to specific target cells or tissues. MEDI-579 is effective in a mouse model of diabetic kidney disease^47^ – a model demonstrated to be dependent upon the protease-inhibitory activity of PAI-1 alone^48^. The data presented here demonstrates that this protease-inhibitory function of PAI-1 is also fundamental in a mouse model of lupus nephritis.

Further mechanistic characterisation of MEDI-579 will build understanding of the specific role of PAI-1 in a variety of disease settings and may have implications for future design of drugs to this therapeutically relevant serpin.

## Materials and Methods

All methods were carried out in accordance with relevant guidelines and regulations.

### Isolation of anti-PAI-1 antibody

PAI-1 antigens were immobilised at 10 µg/ml on Amino Immobiliser plates (Nunc). PAI-1-specific scFvs were isolated from large scFv human antibody library after two rounds of selection essentially as described previously^26^.

### Antibody optimisation by ribosome display

A ribosome-display scFv library was created by random mutagenesis using the Diversify™ PCR Random Mutagenesis Kit (BD Biosciences). The assembled scFv library with gene III tether was expressed *in vitro* using the RiboMAX™ Large Scale RNA Production System (T7) (Promega), following the manufacturers protocol, and an *E*.*coli*- based prokaryotic cell-free translation system. The scFv antibody-ribosome-mRNA (ARM) complexes were incubated in solution with biotinlyated active or mutant human PAI-1 as described previously^49^.

### PAI-1/tPA competition binding assays

Maxisorp plates (Nunc) were coated with 0.5 µg/ml human or rat tPA. Wells were washed PBS + 0.05% (v/v) Tween-20 and blocked with PBS + 3% (w/v) autoclaved dried milk protein. To each well, 25 µl of crude periplasmic scFv and 25 µl of biotinylated human (2 nM) or rat (3 nM) PAI-1 was added and allowed to equilibrate. Binding of PAI-1 was detected using streptavidin-Europium and DELFIA® assay conditions as per manufacturer’s instructions (Perkin Elmer).

### PAI-1: Vitronectin Binding Assay

Human monomeric vitronectin (Molecular Innovations; MI, US) was coated onto 96 well Maxisorp plates (Nunc) at 0.15 µg/ml in PBS w/o Ca^2+^ and Mg^2+^ (Gibco). After one hour at room temperature wells were washed three times with PBS + 0.05% (v/v) Tween-20 (PBS-T) and blocked with PBS + 3% (w/v) skimmed milk powder for a further hour. To each sample well, biotinylated human active PAI-1 (0.5 nM) plus a dilution series of either MEDI-579, 33B08 IgG or an irrelevant isotype-matched IgG control (CAT002 hIgG1κ) pre-mixed at room temperature in PBS + 1% (w/v) skimmed milk powder, was added. Plates were incubated for two hours at room temperature and washed three times with PBS-T. Streptavidin-Europium and DELFIA® assay conditions were followed as per manufacturer’s instructions (Perkin Elmer) to detect biotinylated PAI-1/vitronectin complexes. Plates were read on an EnVision plate reader (Perkin Elmer) using a Europium DELFIA® detection protocol.

### *In vitro* pharmacology assay

Normal human lung fibroblasts (NHLF) or mouse MLg lung fibroblast cells were plated (~20000/well, 96 well format; Lonza, Basel, Switzerland) and stimulated with rhTGFβ (100 pM; R&D Systems, Abingdon, UK) for 18 h to induce the expression of PAI-1. Following washing of cells with HBSS, plasmin generation was then measured by incubation of the cells in phenol red-free HBSS with human lysine-plasminogen (5 μg/ml; Technoclone, Vienna, Austria) and the chromogenic plasmin substrate S2251 (0.2 mM; Chromogenix, Milan, Italy) in the presence or absence of MEDI-579. An irrelevant isotype-matched IgG control (CAT002 hIgG1κ) was included in the NHLF experiments. An increase in absorbance at 405 nm at 37 °C was then followed for up to 3 h.

### Affinity determination

Surface plasmon resonance measurements using a BIAcore T100 (GE Healthcare Life Sciences) were performed essentially as described by Karlsson *et al*.^50^. In brief, antibodies were coupled to CM3 sensorchips using an amine coupling kit (GE Healthcare Life Sciences) at a surface density of approximately 500 RU and a serial dilution (between 6.25 nM and 25 pM) of glycosylated human PAI-1 (and rat PAI-1 (both Molecular Innovations) in HBS-EP + (GE Healthcare Life Sciences) was passed over the sensorchip surface. The resulting sensorgrams were evaluated using the Biacore T100 Evaluation software (version 2.0.3, GE Healthcare Life Sciences) to provide kinetic data.

### Crystallisation and structural determination of the MEDI-579:PAI-1 complex

MEDI-579 IgG_1_ was digested in a solution containing 30 mM DL-cysteine hydrochloride dissolved in PBS (Gibco) plus 1 mg per 100 mg IgG of papain from papaya latex (Sigma). The digest was terminated after 4.5 hours by the addition of 0.5 M iodoacetamide (Sigma) to give 50 mM iodoacetamide in the final digest mixture. MEDI-579 Fab was purified using Q Sepharose (GE Healthcare), buffer exchanged into PBS (Gibco) pH 7.2 and concentrated to 10 mg/ml. Purified protein of a stable quadruple mutant of PAI-1^51^ was obtained from Jim Huntington, University of Cambridge, and produced according to the protocol referred to in Zhou *et al*.^9^. MEDI-579 Fab and PAI-1 were mixed at a 1:1 ratio, followed by buffer exchange into 15 mM ammonium acetate pH 7.4 and concentrated to a final protein concentration of 3–9 mg/ml. Crystals were grown from hanging drops equilibrated against a solution of 8–10% PEG 8000, 0.2 M LiCl and 20% ethylene glycol, then harvested and flash frozen. The initial screening for anisotropically diffracting and non-twinned crystals was carried out at the beamline 911:2 at the MAX IV Laboratory. Crystals were stored in preparation for data collection at the European Synchrotron Radiation Facility in Grenoble. A second data set to 2.9 Å, used for the final refinement, was collected at beamline ID29 (wavelength of 0.976). Data were processed with MOSFLM^52^ and scaled with SCALA from the CCP4 suite^53^. The structure was determined by molecular replacement using the MOLREP^54^. Search models were obtained from a crystal structure of PAI-1 stability mutant (PDB code 4g8r)^21^ and a crystal structure of a Fab (PDB code 1aqk)^55^. Manual rebuilding of the model, which has four copies of a 1:1 complex of PAI-1 and Fab, was performed using the model-building program COOT^56^. The model was refined to convergence using Autobuster^57^. In the final model 95% of the residues were in favoured regions of the Ramachandran plot, with 4% in additional allowed regions.

### Mouse lupus nephritis model

All procedures were performed in accordance with federal, state and Institutional guidelines in an AAALAC-accredited facility and were approved by the MedImmune Institutional Animal Care and Use Committee.

On day 0, mice were injected intravenously (lateral tail vein) with 0.3 × 10^10^ viral particles of either Ad Null (empty vector control) or Adenovirus-interferon-alpha (Adv-IFN) in 0.1 M phosphate buffered saline (PBS) pH 7.2 (Gibco). Twice weekly dosing of MEDI-579 (1 mg/kg or 10 mg/kg; or antibody control, 10 mg/kg, intraperitoneally) began immediately following adenovirus delivery on day 0 and continued throughout the course of the study. Mice were placed in metabolic cages once weekly in order to collect 24 hour urine samples. These urine samples were frozen and sent to AniLytics (Gaithersburg, MD) for chemistry analysis; total protein, creatinine and sodium. For the single dose pharmacodynamic assessment, NZBxNZW/F1 mice that had a dipstick reading of greater than 1 + (Chemstrip 2 GP, Roche, Indianapolis IN) at four weeks post-Adv-IFN infection, received a single dose of MEDI-579 (10 mg/kg; or antibody control, 10 mg/kg, intraperitoneally). Mice were terminated 48 hours following administration and MEDI-579; active PAI-1 and active plasmin were measured in plasma.

### Assessment of Active PAI-1 and Plasmin

Citrated plasma was collected by collecting whole blood into Eppendorf tubes containing 3.2% sodium citrate (1/10 the total sample volume). The blood was then centrifuged, plasma was drawn off, put into a clean Eppendorf tube and centrifuged again, samples were then frozen until assayed. Right kidneys collected, halved and frozen in liquid nitrogen until homogenised in ice-cold neutral lysis buffer (1% Triton X-100, 1 mM EDTA and protease inhibitors in PBS pH 7.2). The lysates were centrifuged and supernatants were transferred to new tubes and frozen until assayed. Mouse active PAI-1 and mouse active plasmin ELISA kits were purchased from Innovative Research (Nova, Michigan). Manufacturer instructions were followed for all assays.

### Accession code

Coordinates and structure factors have been deposited in the Protein Data Bank, with accession code 6I8S.

## Supplementary information

Supplementary Information

## References

[1] Pannekoek H (1986). Endothelial plasminogen activator inhibitor (PAI): a new member of the Serpin gene family. The EMBO journal.

[2] Dupont DM (2009). Biochemical properties of plasminogen activator inhibitor-1. Frontiers in bioscience (Landmark edition).

[3] Gils A, Declerck PJ (2004). The structural basis for the pathophysiological relevance of PAI-I in cardiovascular diseases and the development of potential PAI-I inhibitors. Thrombosis and haemostasis.

[4] Kruithof EK, Tran-Thang C, Bachmann F (1986). The fast-acting inhibitor of tissue-type plasminogen activator in plasma is also the primary plasma inhibitor of urokinase. Thrombosis and haemostasis.

[5] Stefansson S, Lawrence DA (1996). The serpin PAI-1 inhibits cell migration by blocking integrin alpha V beta 3 binding to vitronectin. Nature.

[6] Kjoller, L. *et al*. Plasminogen activator inhibitor-1 represses integrin- and vitronectin-mediated cell migration independently of its function as an inhibitor of plasminogen activation. *Experimental cell research* **232**, 420–429, doi:S0014-4827(97)93540-0 [pii] (1997). 10.1006/excr.1997.3540 9168821

[7] Lin Z (2011). Structural basis for recognition of urokinase-type plasminogen activator by plasminogen activator inhibitor-1. The Journal of biological chemistry.

[8] Gong L (2015). Crystal Structure of the Michaelis Complex between Tissue-type Plasminogen Activator and Plasminogen Activators Inhibitor-1. The Journal of biological chemistry.

[9] Zhou A, Huntington JA, Pannu NS, Carrell RW, Read RJ (2003). How vitronectin binds PAI-1 to modulate fibrinolysis and cell migration. Nature structural biology.

[10] Hekman CM, Loskutoff DJ (1985). Endothelial cells produce a latent inhibitor of plasminogen activators that can be activated by denaturants. The Journal of biological chemistry.

[11] Declerck PJ, De Mol M, Vaughan DE, Collen D (1992). Identification of a conformationally distinct form of plasminogen activator inhibitor-1, acting as a noninhibitory substrate for tissue-type plasminogen activator. The Journal of biological chemistry.

[12] Mottonen J (1992). Structural basis of latency in plasminogen activator inhibitor-1. Nature.

[13] Sharp AM (1999). The active conformation of plasminogen activator inhibitor 1, a target for drugs to control fibrinolysis and cell adhesion. Structure (London, England : 1993).

[14] Nar H (2000). Plasminogen activator inhibitor 1. Structure of the native serpin, comparison to its other conformers and implications for serpin inactivation. Journal of Molecular Biology.

[15] Aertgeerts K, De Bondt HL, De Ranter CJ, Declerck PJ (1995). Mechanisms contributing to the conformational and functional flexibility of plasminogen activator inhibitor-1. Nature structural biology.

[16] Debrock S, Declerck PJ (1997). Neutralization of plasminogen activator inhibitor-1 inhibitory properties: identification of two different mechanisms. Biochimica et biophysica acta.

[17] Fjellstrom O (2013). Characterization of a small molecule inhibitor of plasminogen activator inhibitor type 1 that accelerates the transition into the latent conformation. The Journal of biological chemistry.

[18] Lin Z (2013). Structural Insight into Inactivation of Plasminogen Activator Inhibitor-1 by a Small-Molecule Antagonist. Chemistry & biology.

[19] Verhamme I (1999). Accelerated conversion of human plasminogen activator inhibitor-1 to its latent form by antibody binding. The Journal of biological chemistry.

[20] Gorlatova NV, Elokdah H, Fan K, Crandall DL, Lawrence DA (2003). Mapping of a conformational epitope on plasminogen activator inhibitor-1 by random mutagenesis. Implications for serpin function. The Journal of biological chemistry.

[21] Li SH (2013). Mechanistic characterization and crystal structure of a small molecule inactivator bound to plasminogen activator inhibitor-1. Proceedings of the National Academy of Sciences of the United States of America.

[22] Wind T, Jensen MA, Andreasen PA (2001). Epitope mapping for four monoclonal antibodies against human plasminogen activator inhibitor type-1: implications for antibody-mediated PAI-1-neutralization and vitronectin-binding. European journal of biochemistry/FEBS.

[23] Gils A (2003). Biochemical importance of glycosylation of plasminogen activator inhibitor-1. Thrombosis and haemostasis.

[24] Bijnens AP (2001). Elucidation of the binding regions of PAI-1 neutralizing antibodies using chimeric variants of human and rat PAI-1. Thrombosis and haemostasis.

[25] Novoa de Armas H, Dewilde M, Verbeke K, De Maeyer M, Declerck PJ (2007). Study of recombinant antibody fragments and PAI-1 complexes combining protein-protein docking and results from site-directed mutagenesis. Structure (London, England : 1993).

[26] Vaughan TJ (1996). Human antibodies with sub-nanomolar affinities isolated from a large non-immunized phage display library. Nature biotechnology.

[27] Lloyd C (2009). Modelling the human immune response: performance of a 1011 human antibody repertoire against a broad panel of therapeutically relevant antigens. Protein engineering, design & selection : PEDS.

[28] Hanes J, Jermutus L, Pluckthun A (2000). Selecting and evolving functional proteins in vitro by ribosome display. Methods in enzymology.

[29] Jermutus L, Honegger A, Schwesinger F, Hanes J, Pluckthun A (2001). Tailoring in vitro evolution for protein affinity or stability. Proceedings of the National Academy of Sciences of the United States of America.

[30] Osada H, Yamada C, Miwa K, Kono T, Oh-hira M (1991). An assay system for the modulators of plasminogen activation on the cell surface. Thrombosis research.

[31] Weisberg, A. D. *et al*. Pharmacological inhibition and genetic deficiency of plasminogen activator inhibitor-1 attenuates angiotensin II/salt-induced aortic remodeling. *Arteriosclerosis*, *Thrombosis*, *and Vascular Biology* **25**, 365–371, doi:01.ATV.0000152356.85791.52 [pii] (2005). 10.1161/01.ATV.0000152356.85791.52 15576638

[32] Oda, T. *et al*. PAI-1 deficiency attenuates the fibrogenic response to ureteral obstruction. *Kidney international* **60**, 587–596, doi:kid846 [pii] (2001). 10.1046/j.1523-1755.2001.030002587.x 11473641

[33] Chuang-Tsai, S. *et al*. Reduction in fibrotic tissue formation in mice genetically deficient in plasminogen activator inhibitor-1. *The American journal of pathology* **163**, 445–452, doi:S0002-9440(10)63674-7 [pii] (2003). 10.1016/S0002-9440(10)63674-7 PMC1868204 12875966

[34] Kaikita K (2001). Plasminogen activator inhibitor-1 deficiency prevents hypertension and vascular fibrosis in response to long-term nitric oxide synthase inhibition. Circulation.

[35] Bergheim, I., Guo, L., Davis, M. A., Duveau, I. & Arteel, G. E. Critical role of plasminogen activator inhibitor-1 in cholestatic liver injury and fibrosis. *The Journal of pharmacology and experimental therapeutics* **316**, 592–600, doi:jpet.105.095042 [pii] (2006). 10.1124/jpet.105.095042 16221737

[36] Lopez-Guisa JM, Rassa AC, Cai X, Collins SJ, Eddy AA (2011). Vitronectin accumulates in the interstitium but minimally impacts fibrogenesis in experimental chronic kidney disease. American journal of physiology.Renal physiology.

[37] Moll S (1995). Induction of plasminogen activator inhibitor type 1 in murine lupus-like glomerulonephritis. Kidney international.

[38] Jensen JK (2011). Crystal structure of plasminogen activator inhibitor-1 in an active conformation with normal thermodynamic stability. The Journal of biological chemistry.

[39] Huntington JA (2011). Serpin structure, function and dysfunction. Journal of thrombosis and haemostasis : JTH.

[40] Fortenberry YM (2013). Plasminogen activator inhibitor-1 inhibitors: a patent review (2006-present). Expert opinion on therapeutic patents.

[41] Capra JD, Kehoe JM (1974). Variable region sequences of five human immunoglobulin heavy chains of the VH3 subgroup: definitive identification of four heavy chain hypervariable regions. Proceedings of the National Academy of Sciences of the United States of America.

[42] Raghunathan G, Smart J, Williams J, Almagro JC (2012). Antigen-binding site anatomy and somatic mutations in antibodies that recognize different types of antigens. Journal of Molecular Recognition : JMR.

[43] Deng G, Curriden SA, Wang S, Rosenberg S, Loskutoff DJ (1996). Is plasminogen activator inhibitor-1 the molecular switch that governs urokinase receptor-mediated cell adhesion and release?. J Cell Biol.

[44] Bajou K (2001). The plasminogen activator inhibitor PAI-1 controls in vivo tumor vascularization by interaction with proteases, not vitronectin. Implications for antiangiogenic strategies. The Journal of cell biology.

[45] Courey AJ (2011). The vitronectin-binding function of PAI-1 exacerbates lung fibrosis in mice. Blood.

[46] Zhong J (2014). Vitronectin-binding PAI-1 protects against the development of cardiac fibrosis through interaction with fibroblasts. Laboratory investigation; a journal of technical methods and pathology.

[47] Gu C, Zhang J, Noble NA, Peng XR, Huang Y (2016). An additive effect of anti-PAI-1 antibody to ACE inhibitor on slowing the progression of diabetic kidney disease. American journal of physiology. Renal physiology.

[48] Huang Y, Border WA, Lawrence DA, Noble NA (2009). Mechanisms underlying the antifibrotic properties of noninhibitory PAI-1 (PAI-1R) in experimental nephritis. American journal of physiology.Renal physiology.

[49] Thom G (2006). Probing a protein-protein interaction by in vitro evolution. Proceedings of the National Academy of Sciences of the United States of America.

[50] Karlsson R, Michaelsson A, Mattsson L (1991). Kinetic analysis of monoclonal antibody-antigen interactions with a new biosensor based analytical system. Journal of immunological methods.

[51] Berkenpas MB, Lawrence DA, Ginsburg D (1995). Molecular evolution of plasminogen activator inhibitor-1 functional stability. The EMBO journal.

[52] Leslie, A. G. W. & Powell, H. R. In *Evolving Methods for Macromolecular* Crystallography (eds Read, R. J. & Sussman, J. L.) 41 (Springer, 2007).

[53] Collaborative Computational Project, N (1994). The CCP4 suite: programs for protein crystallography. Acta crystallographica.Section D, Biological crystallography.

[54] Vagin A, Teplyakov A (2010). Molecular replacement with MOLREP. Acta crystallographica.Section D, Biological crystallography.

[55] Faber C (1998). Three-dimensional structure of a human Fab with high affinity for tetanus toxoid. Immunotechnology : an international journal of immunological engineering.

[56] Emsley P, Cowtan K (2004). Coot: model-building tools for molecular graphics. Acta crystallographica.Section D, Biological crystallography.

[57] Bricogne, G. *et al*. (Global Phasing Limited, Cambridge, UK, 2011).

[58] Jensen JK, Gettins PG (2008). High-resolution structure of the stable plasminogen activator inhibitor type-1 variant 14-1B in its proteinase-cleaved form: a new tool for detailed interaction studies and modeling. Protein science : a publication of the Protein Society.

[59] Huntington JA, Read RJ, Carrell RW (2000). Structure of a serpin-protease complex shows inhibition by deformation. Nature.

