# Supplementary material for: Discovery and characterisation of an antibody that selectively modulates the inhibitory activity of plasminogen activator inhibitor-1
Source: Sci Rep. 2019 Feb 7;9:1605. doi: 10.1038/s41598-019-38842-x (PMC6367345; doi:10.1038/s41598-019-38842-x)
Supplement: Supplementary file 1 — Supplementary Information [file 41598_2019_38842_MOESM1_ESM.pdf]

SUPPLEMENTARY INFORMATION

Discovery and characterisation of an antibody that selectively modulates the inhibitory activity of plasminogen activator inhibitor-1

Katherine A. Vousden, Tomas Lundqvist, Bojana Popovic, Brian Naiman, Alan M. Carruthers, Philip Newton, Daniel J. D. Johnson, Anja Pomowski, Trevor Wilkinson, Patrick Dufner, Isabelle de Mendez, Philip R. Mallinder, Clare Murray, Martin Strain, Jane Connor, Lynne A. Murray, Matthew A. Sleeman, David C. Lowe, James A. Huntington, Tristan J. Vaughan

SUPPLEMENTARY METHODS

Recombinant PAI-1 and protease reagents

Unless otherwise stated, all recombinant proteins were *E.coli* expressed. Unless otherwise stated, any experiments performed using human PAI-1 refers to the use of human active PAI-1 from Molecular Innovations Inc. Biotinylation of recombinant PAI-1 was performed using EZ link NHS-LC-Biotin (Pierce Protein Research products; Thermo Fisher Scientific, Northumberland, UK). PAI-1 and tPA reagents used in antibody selection and *in vitro* assays are in Supplementary Table S1.

tPA-coupled cell-free chromogenic assays. Assays were carried out at room temperature in 96-well assay plates (Costar) in 50 µl assay buffer (100 mM Tris pH 8.4, 106 mM NaCl, 0.01% Tween 80). Recombinant human (5 nM), rat (2 nM) or mouse (6 nM) PAI-I were incubated with purified scFvs for 10 mins. Human tPA (2.14 nM; Actilyse, Boehringer Ingelheim), rat tPA (1.34 nM) or mouse tPA (1.63 nM; both Molecular Innovations Inc) were added and the samples incubated for a further 5 minutes. Chromogenic substrate (S2288, Quadratech) was added to the human (920 µM), rat (414 µM) and mouse (452 µM) assays. After 1 hour 15 minutes incubation, OD 405 nm was measured using an EnVision plate reader (PerkinElmer).

Analysis of MEDI-579 PAI-1 complex formation and activity

Stable native PAI-1 or latent PAI-1 (0.25 mg) (Molecular Innovations) was incubated with 1.5 mg MEDI-579 (2 fold molar excess of antibody) at room temperature for 30 minutes. Size exclusion chromatography was carried out using a Superdex 200 10/300 GL column (GE Healthcare Life Sciences) equilibrated in 50 mM Tris pH 7.4, 150 mM NaCl. The sample was loaded and the column run at 0.5 ml/min and 0.5 ml fractions collected (Supplementary Figure S2b). Samples from each fraction of the latent PAI-1/MEDI-579 experiment were run under non-reducing conditions on a 10 % Bolt Bis Tris SDS PAGE gel (ThermoFisher Scientific) and stained with Quick Coomassie stain (Generon). The bands corresponding to PAI-1 (either complexed with MEDI-579, fractions B9-B14, or un-complexed, fractions C2-C6) were quantified by densitometry on a Typhoon scanner (GE Healthcare Life Sciences) (Supplementary Figure S2c). To determine whether latent PAI-1 contained any native PAI-1, 2 µg latent PAI was reacted with 3.2 µg thrombin (Cambridge Protein Works) for 30 minutes in the presence or absence of 3.0 µM heparin. Cleavage in the reactive centre loop is an indication of the presence of native PAI-1 and is indicated by the appearance of a new band on SDS PAGE immediately below the PAI-1 band. Latent PAI-1 that did not form a complex with MEDI-579 (pool of fractions C1 to C6 from the gel

filtration run of latent PAI-1 with MEDI-579) was treated in the same way. After incubation with thrombin the samples were boiled in non-reducing SDS sample buffer and run on an SDS PAGE gel and stained with Coomassie blue (Supplementary Figure S2d).

Competition ELISA

Latent human PAI-1 (Molecular Innovations), human PAI-2 and PAI-3 (J Huntington), α1-anti-trypsin and antithrombin III (US Biological), α-1 antichymotrypsin (Research Diagnostics Inc.) and α1-antiplasmin (American Diagnostica) were used in competition ELISAs. MEDI-579 was coated on a Maxisorp plate (Nunc) at 0.3 µg/ml in PBS. Wells were washed three times with 300 µl of PBS + 0.05% Tween-20 and blocked with 200 µl/well of PBS + 3% (w/v) dried milk protein for one hour at room temperature. To each well, 25 µl of competitor antigen prepared as a log3 dilution series in PBS from a top concentration of 1 µM, plus 25 µl of biotinylated human active PAI-1 at 1 nM was added and allowed to equilibrate for 1 hour at room temperature. Following three wash cycles as before, binding of biotinylated human PAI-1 was detected using streptavidin-Europium and DELFIA® assay conditions as per manufacturer's instructions (Perkin Elmer). Plates were read on an EnVision plate reader (Perkin Elmer) using a Europium detection protocol.

SUPPLEMENTARY TABLE

Table S1. Recombinant PAI-1 and tPA reagents

**Reagent**	**Source**
Human active PAI-1	Molecular Innovations Inc (Novi MI, US)
Human PAI-1 (14-1B)	Molecular Innovations Inc (Novi MI, US)
Mouse PAI-1	Molecular Innovations Inc (Novi MI, US)
Rat PAI-1	US Biological (Salem MA, US)
Human PAI-1 (CHO expressed)	AstraZeneca
Human tPA (Actilyse)	Boehringer Ingelheim (Ingelheim am Rhein, Germany)
Rat tPA	Molecular Innovations Inc (Novi MI, US)
Mouse tPA	Molecular Innovations Inc (Novi MI, US)

SUPPLEMENTARY FIGURES

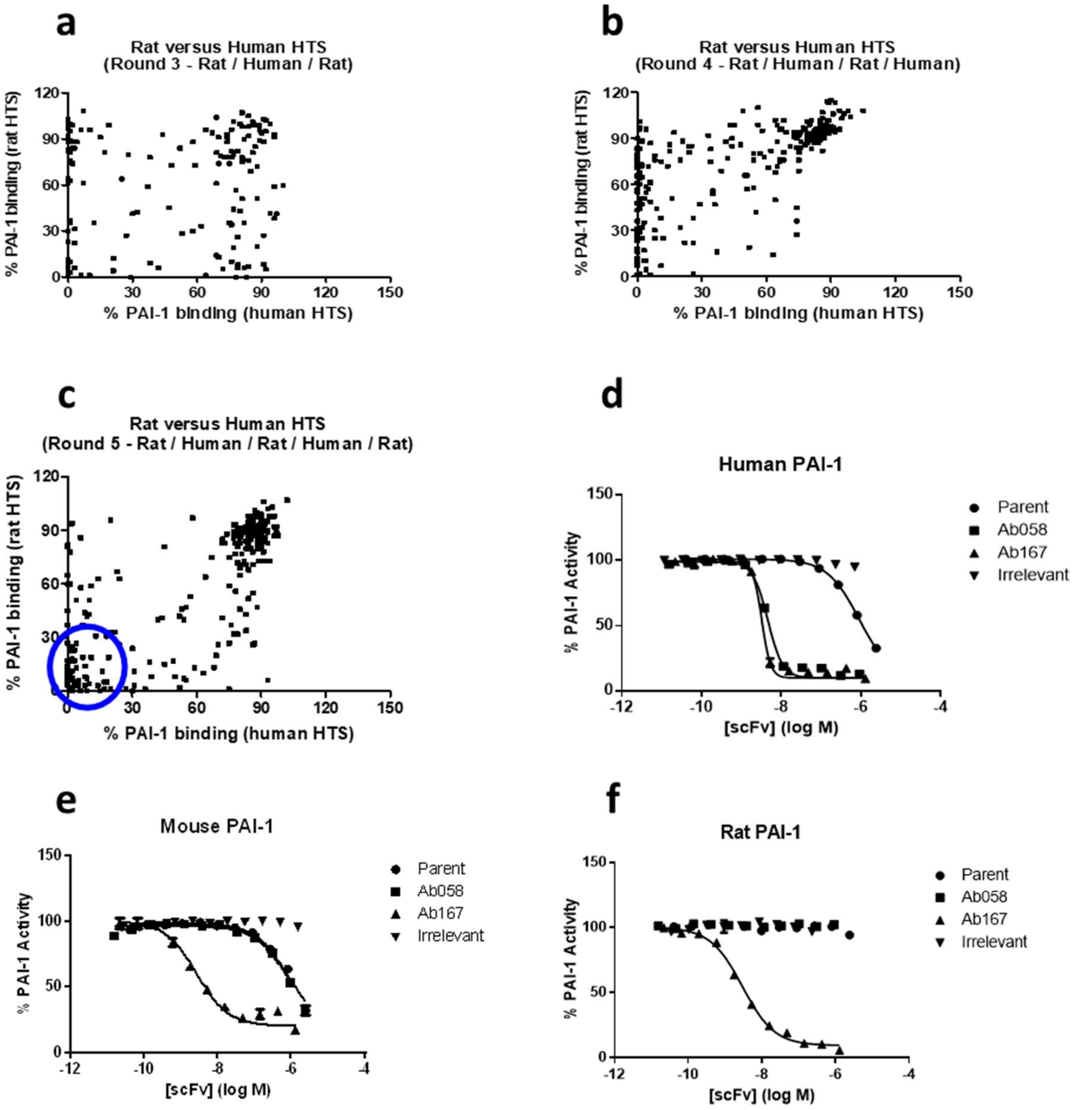

Figure S1: Positive selection for rodent cross-reactivity. Correlation analysis between human and rat PAI-1/tPA competition assay periplasmic scFv screening data after (**a**) three (**b**) four and (**c**) five rounds of selection. Circled in panel (c) is the appearance of human/rat cross-reactive scFv's enriched after five rounds of selection with alternating human and rat PAI-1 antigens. (**d-f**) Purified scFv profiling in tPA-coupled cell-free chromogenic assays. Ab058 was identified after three rounds of selection on human PAI-1 only. Ab167 was identified from the high-throughput screen depicted in (c).

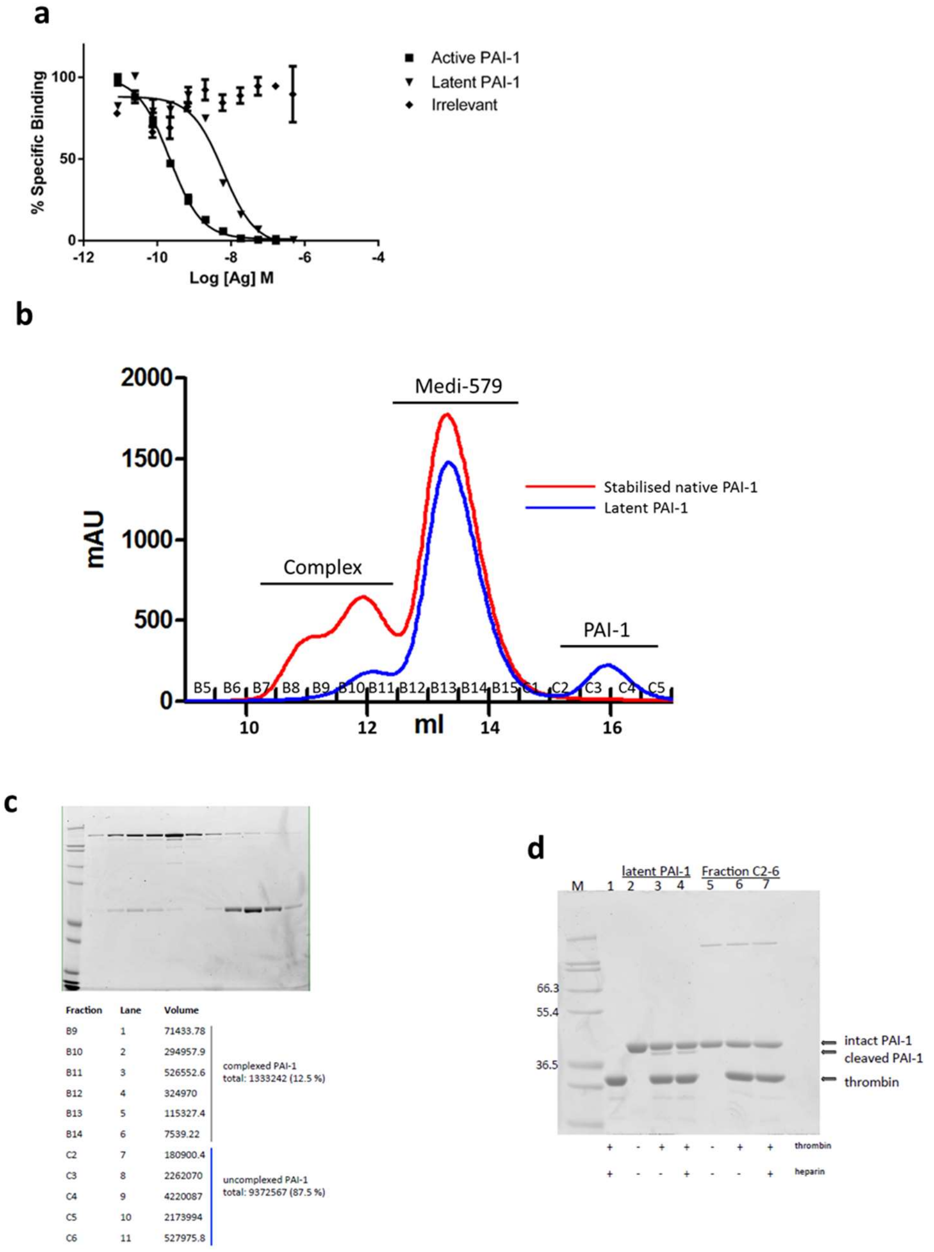

Figure S2. MEDI-579 specifically binds active PAI-1. (**a**) Commercially sourced latent PAI-1 is able to compete with active PAI-1 for MEDI-579 binding, with an approximate 10-fold loss of affinity compared to unlabelled active PAI-1. (**b**) MEDI-579/PAI-1 complexes were formed using both stabilised native and a commercial source of latent PAI-1 and assessed by size exclusion chromatography (SEC). Complex formation was achieved with both active (red) and latent (blue) sources of PAI-1. (**c**) MEDI-579/latent PAI-1 complex formation was quantified by typhoon densitometry and fractions separated. Uncomplexed PAI-1, represents approximately 87.5 % of the starting sample. High MW bands are MEDI-579. Low MW bands are PAI-1. (**d**) Uncomplexed PAI-1 (Fractions C2-6; lanes 5-7) and original latent PAI-1 preparation (lanes 2-4) were screened for activity by thrombin cleavage assay in the presence or absence of heparin. Uncomplexed PAI-1 fractions were shown to be inactive, whereas the original latent PAI-1 preparation retains some activity as evidenced by the production of cleaved PAI-1 in the presence of thrombin. We hypothesise that MEDI-

579/PAI-1 complexes formed using latent PAI-1 preparations is due to the presence of active PAI-1 in these samples.

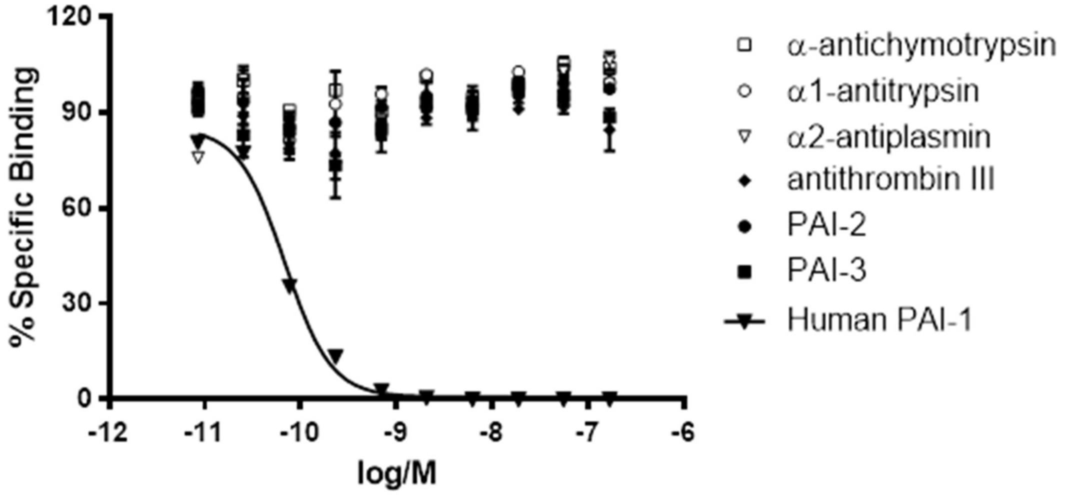

Figure S3. MEDI-579 is selective for PAI-1 over other serpins. Human PAI-1, but not related serpins, is able to compete for MEDI-579 binding to labelled human PAI-1. Data shown are representative of two independent experiments.

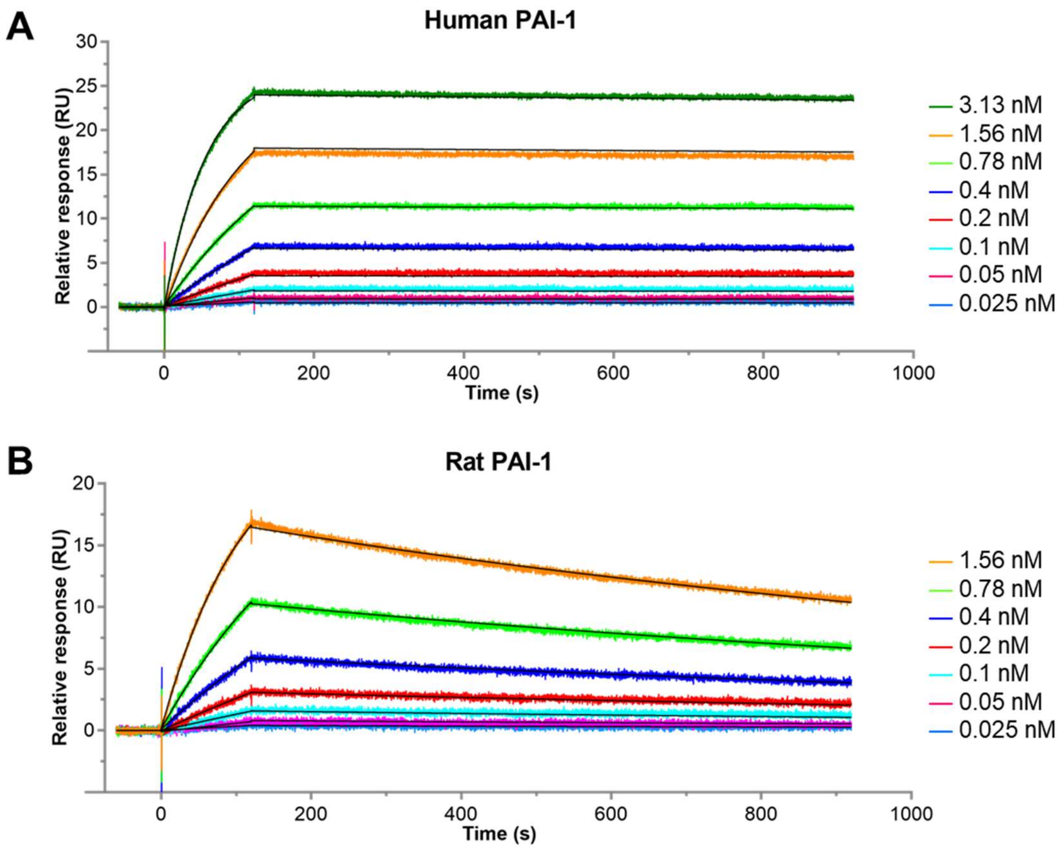

Figure S4: Example BIAcore kinetic analysis. (A) Human PAI-1 (ka = 5.88E+06 1/Ms, kd = 3.14E-05 1/s, KD = 5.34 pM, chi2 = 0.088 RU2) and (B) Rat PAI-1 (ka = 8.01E+06 1/Ms, kd = 6.73E-04 1/s, KD = 84 pM, chi2 = 0.028 RU2) binding to MEDI-579 Fab using a 1:1 binding model.

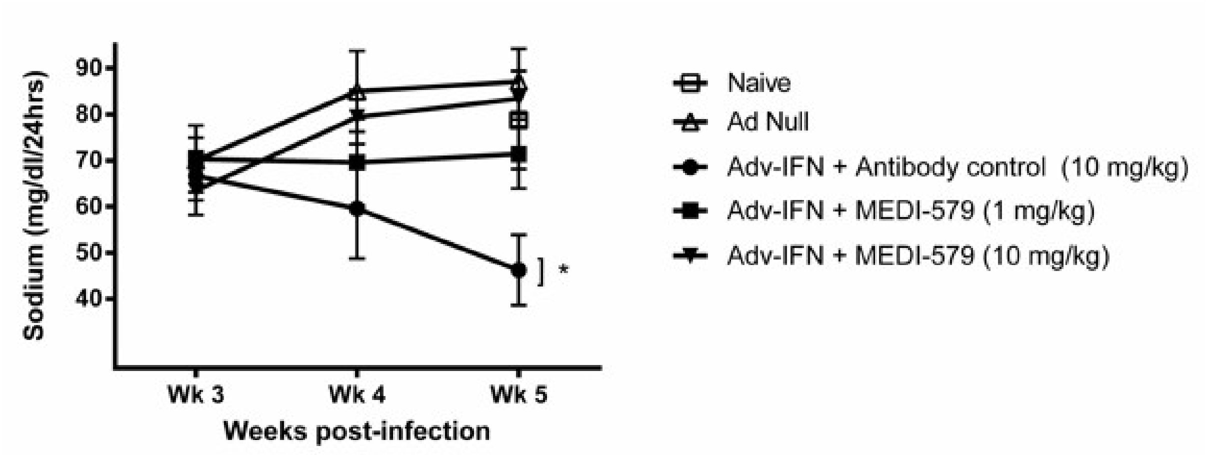

Figure S5. MEDI-579 treatment normalizes urinary sodium output at the 10mg/kg dose. Urinary sodium levels were obtained from urine samples collected over a 24 hour period; n=12 mice/group, shown as group average ± SEM. Antibody control treated mice had significantly lower urinary sodium output compared to MEDI-579 (10 mg/kg) treated mice, indicating that the MEDI-579 (10 mg/kg) treatment group maintained normal kidney function; *P<0.05 MEDI-579 (10 mg/kg) compared to antibody control at Wk 5, Kruskal-Wallis with Dunn's multiple comparison.

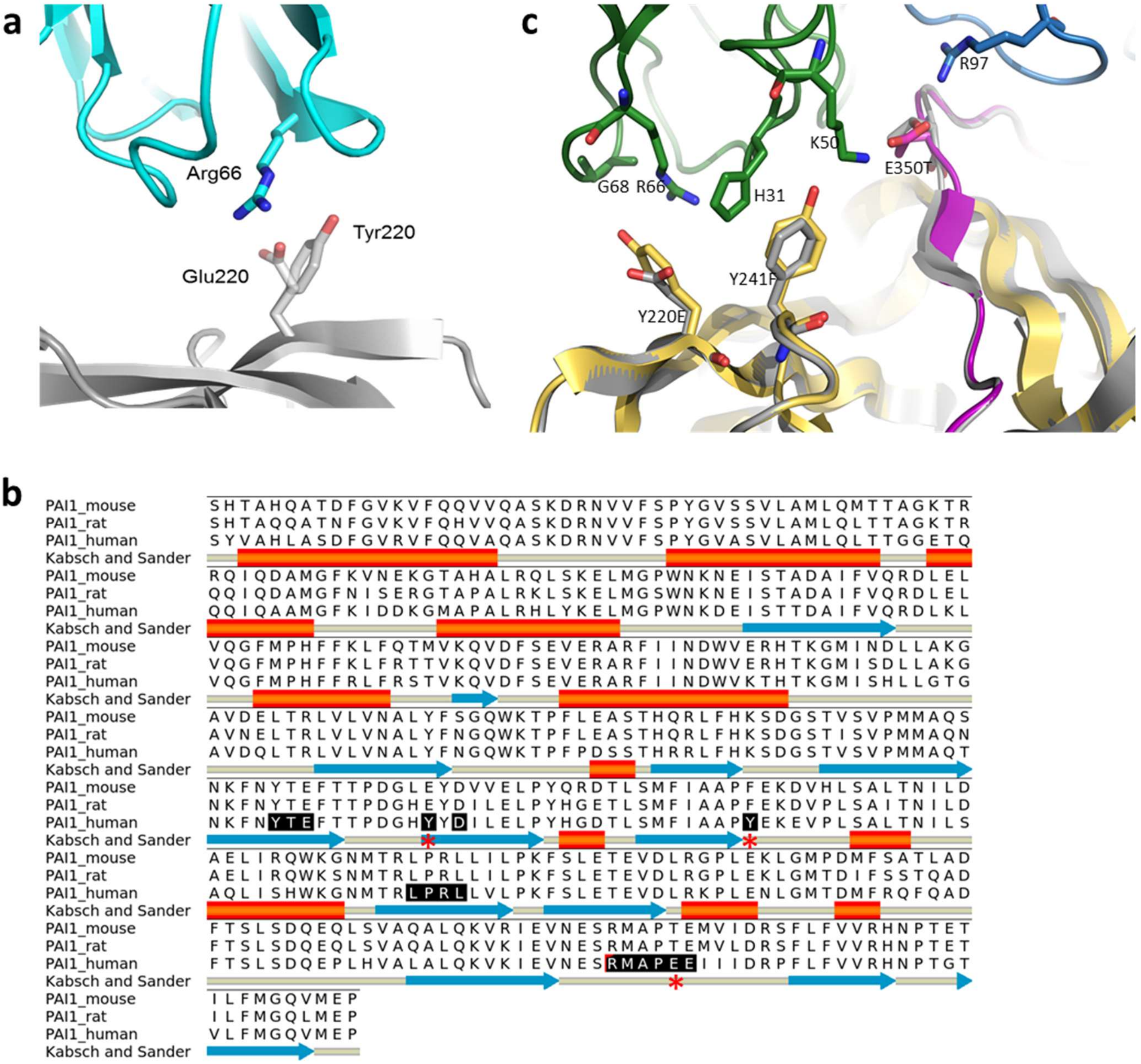

Figure S6. Comparison of human and rodent PAI-1:MEDI-579 interactions (a) A homology model of rat PAI-1 was built using human active PAI-1 as a template (PDB code 1DVM [1]). PAI-1 shown in grey; MEDI-579 VL is blue. Arg66 of MEDI-579 light chain is predicted to form a salt bridge with rat PAI-1 Glu220, and makes favourable π-staking interactions with human PAI-1 Tyr220. (**b**) Human, mouse and rat PAI-1 alignment. Black shading indicates PAI-1 residues within the MEDI-579 epitope. Three sequence differences between rodent and human PAI-1 within the epitope (human positions first): Tyr220Glu, Tyr241Phe and Glu350Thr are indicated with a red asterisk. These amino acid substitutions are identical in mouse and rat PAI-1 sequences. (**c**) A homology model of rat PAI-1 was built using human active PAI-1 as a template (PDB code 1DVM [1]). MEDI-579 (VH blue, VL green), human PAI-1 (yellow) and rat PAI-1 (grey).

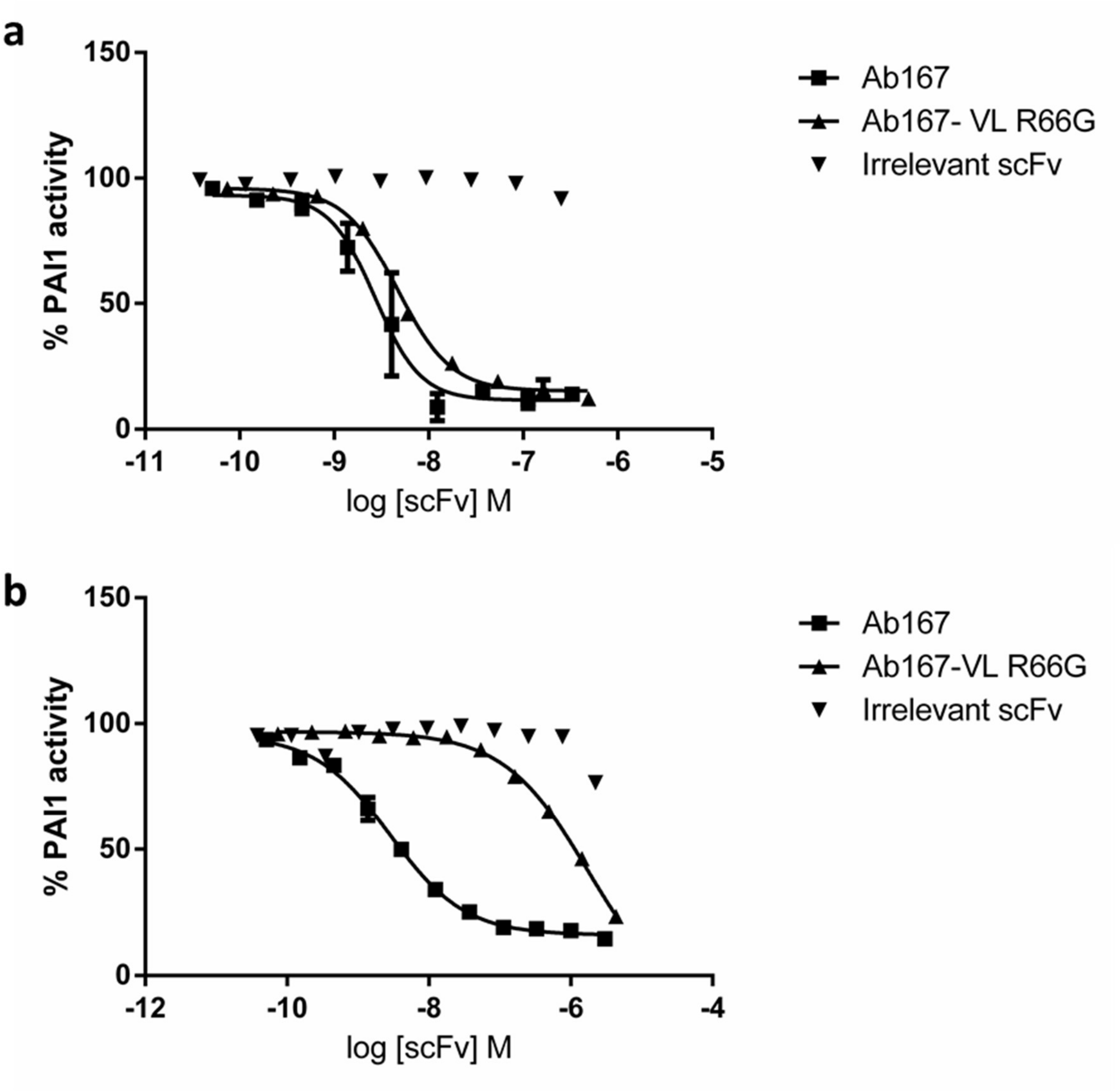

Figure S7. VL R66 important for rat cross-reactivity. (**a**) Ab167 scFv (MEDI-579) and Ab167-VL R66G scFv are potent in human PAI-1 chromogenic assay. (**b**) Ab167 scFv is significantly more potent that Ab167 R66G in a rat chromogenic assay.

SUPPLEMENTARY REFERENCES

1 Stout, T.J., Graham, H., Buckley, D.I. & Matthews, D.J. Structures of active and latent PAI-1: a possible stabilizing role for chloride ions. *Biochemistry* **39**, 8460-8469 (2000).
